# Evaluation of the Deadly Liver Mob program: insights for roll-out and scale-up of a pilot program to engage Aboriginal Australians in hepatitis C and sexual health education, screening, and care

**DOI:** 10.1186/s12954-018-0209-y

**Published:** 2018-02-01

**Authors:** Carla Treloar, Max Hopwood, Elena Cama, Veronica Saunders, L. Clair Jackson, Melinda Walker, Catriona Ooi, Ashley Ubrihien, James Ward

**Affiliations:** 1 Centre for Social Research in Health, UNSW Sydney, Sydney, Australia; 2 Western Sydney Sexual Health Centre, Western Sydney Local Health District, Sydney, Australia; 3 Western Sydney HIV and Related Programs Unit, Western Sydney Local Health District, Sydney, Australia; 4 South Australian Health and Medical Research Institute, Adelaide, Australia

**Keywords:** Aboriginal Australians, Hepatitis C, STIs, Incentives, Implementation

## Abstract

**Background:**

Deadly Liver Mob (DLM) is a peer-driven, incentivised health promotion program aimed at increasing understanding of hepatitis C, promoting harm reduction in relation to injecting drug use, and linking participants to screening for hepatitis C, other blood borne viruses and sexually transmissible infections among Aboriginal people in Western Sydney, NSW. This paper presents the evaluation of a pilot study examining the acceptability of the program as a first step of a scalability assessment.

**Methods:**

Deadly Liver Mob operated in co-located needle and syringe programs and sexual health clinics in two sites: (Site 1: two and a half years for 2 days/week; Site 2: 1 year for 1 day per week). Comparisons were made of the proportion of Aboriginal clients (Site 1) and occasions of service provided to Aboriginal clients (Site 2) in the 12 months prior and post-introduction of DLM. Interviews were conducted with 13 staff involved in delivery of DLM and with 19 clients.

**Results:**

A total of 655 and 55 Aboriginal clients, respectively, attended Site 1 and Site 2 for health education. The proportion of Aboriginal clients attending both sites was significantly higher during the DLM compared with prior to its implementation. Of those attending for health education, 79 and 73%, respectively, attended screening following education. DLM clients strongly endorsed the program. Some staff were concerned about workforce capacity to effectively engage Aboriginal clients with multiple and complex needs, managing the differing aims of the participating services involved, and about offering of incentives for attendance at health services.

**Conclusion:**

While acceptability was high among staff and clients and preliminary results show high engagement with Aboriginal communities, this evaluation of a pilot program raises some issues to consider in scale up of DLM to other sites. The initiation of additional DLM sites should address issues of alignment with governing strategies and workforce capacity.

## Background

Globally, widespread adoption of interventions found to be effective in research or pilot programs is required to achieve population health impact and effect [1]. However, appraisal is required to assess the feasibility of scaling up interventions, including analysis of and assessment of workforce capacity, system and infrastructure requirements, and management and monitoring systems [2]. In research and service delivery with Indigenous populations, research benefit, sustainability, and transferability are key precepts for implementation and are required to avoid disruption of service delivery following closure of research or pilot programs [3]. These issues point to the need for applied research regarding whether and how pilot programs with Indigenous communities can be rolled out to other sites. Deadly Liver Mob (DLM) was a pilot program conducted by mainstream public health services in Western Sydney, Australia, aimed at improving hepatitis C virus (HCV) health promotion, and screening for HCV, HIV, and sexually transmissible infections (STIs) screening among Aboriginal and Torres Strait Islander (hereafter Aboriginal) people. The program was administered in two sites through co-located needle and syringe programs (NSP) and sexual health clinics. This paper reports on exploratory data from a pilot study to examine issues relevant to potential scale-up of this program to additional sites.

Aboriginal Australians comprise approximately 3% of the population and as a group, experience significant disadvantage including life expectancy of approximately 10 years less than non-Aboriginal Australians [4]. Aboriginal people are over represented in Australian profiles of blood-borne viruses (BBVs) and STIs. As a result Aboriginal people are identified as a priority population for respective national and jurisdictional strategies [5, 6]. In Australia, it is estimated that approximately 8–12% of all HCV diagnoses are among Aboriginal people, despite Aboriginal people representing 3% of the total Australian population [7]. Furthermore, over the last 5 years, a divergence in HIV notifications has also occurred between Aboriginal and non-Indigenous Australians, with the current rate of HIV diagnosis more than double that of non-Indigenous Australians; almost 20% of these diagnoses have occurred as a result of injecting drug use [8]. In 2015, Chlamydia, gonorrhoea, syphilis, and HCV among Aboriginal people were diagnosed at 4, 30, 5, and 4 times respectively the rates of non-Aboriginal people in Australia [9]. HCV notifications among Aboriginal people have increased 43% over the last 5 years while rates have remained stable in the non-Aboriginal population [9]. The intersection of these infections and associated behaviours (unsafe sex or injecting drugs) with racism can cause significant shame, stigma, and barriers to engagement with health services [10].

It is imperative to engage Aboriginal people in a range of health services to reduce harms associated with BBVs and STIs and prevent further infections. Publicly funded sexual health services are key providers of care for HCV and other BBVs and STIs but the overall participation of Aboriginal people in these services is low. Data arising from mainstream sexual health services, general practitioners, and Aboriginal Community Controlled Health Services (ACCHS) across Australia show that only 4% of all clients are Aboriginal despite the significant burden of disease in this population [11, 12]. Engaging Aboriginal people within health services can be challenging, particularly young people and people who inject drugs, and this can be attributed to contemporary and historical issues in health service provision, including stigma [13].

Since 2013, the Deadly Liver Mob (DLM) program has been running as a pilot program in one Western Sydney publicly funded, co-located sexual health service and needle and syringe program (NSP), and in a second service in Western Sydney since 2015. The program was conceived to address low attendance at the NSP and sexual health clinic by Aboriginal people in a geographical area with recognized high levels of injecting drug use and low STI screening, and very few episodes of care over the immediate preceding years in the adjacent sexual health clinic, despite being one of the most populous areas for Aboriginal people in Australia.

DLM uses an incentive-based, peer-driven intervention model. This approach is modelled on The Safe Injecting Cwiz (SIC) project which was conducted in Western Sydney in 1998–2002 and targeted people under 25 years of age who injected drugs [14]. The SIC project was an adaptation of a HIV peer-driven intervention for injecting drug users conducted in the USA [15–17], which reached into hidden networks. In DLM, Aboriginal people who inject drugs are invited to an education session with an Aboriginal worker at the NSP after which participants are offered referral to the co-located sexual health service for STI and BBV assessment and screening. The Aboriginal worker accompanies the DLM client to the co-located sexual health service if the client takes up the offer of screening. Sexual health clinic staff manage screening, delivery of results, and provision of treatment (if required) as per standard care. [14–17]

**Fig. 1:**
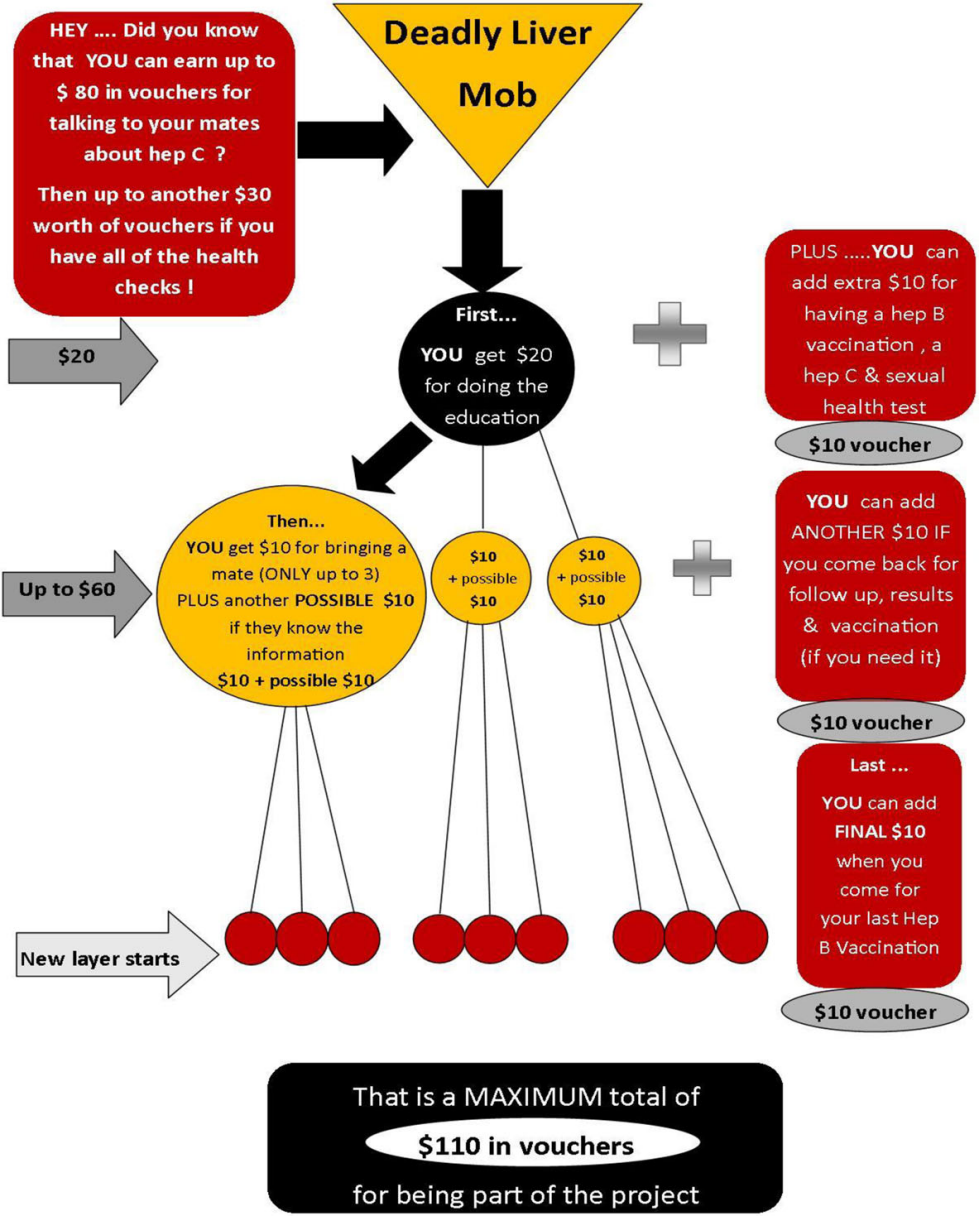
Deadly Liver Mob (DLM) schema for incentive payment in relation to education, peer referral, and screening (poster displayed for DLM clients)

The peer-driven intervention model offers nominal incentives to encourage participants to attend for education then recruit and educate their peers (referred to as peer referral) for which participants receive an incentive (see Fig. 1). People educated in the community by DLM participants then attend the DLM program for consolidation of health messages, receive an “incentive payment,” and are then offered screening. The HCV education sessions monitor the quality of peer messages and build on the participants’ knowledge while dispelling any myths. An additional incentive is offered for HCV and other BBV and sexual health screening and further incentives are offered for return for results and management, as required.

The DLM education model was developed to meet the needs of the audience and to ensure that messages were culturally appropriate. Aboriginal culture is one of sharing stories (or “yarning”) [18] hence the model of talking to participants about HCV and then asking them to pass on the messages to their peers was seen as a culturally effective approach. The HCV education is guided by visual aids and addresses: (1) What is hepatitis C?; (2) How do I get it?; (3) How do I avoid it?; and (4) What can I do if I have it? The education session was designed to be easily understood by participants with low literacy, and to take into account possible negative experiences of education, by remaining non-authoritative and conversational. Although the initial engagement with DLM participants is focussed on HCV education, the program encompasses a broader holistic approach to healthy living [19]. By offering opportunistic screening for HCV, hepatitis B (HBV), HIV, STIs, and providing HBV vaccinations, this population-level approach is designed to reach through family and peer networks and provide a point of entry to other health services.

Aboriginal staff were central to the program design and implementation. A key principle of work with Aboriginal communities is consultation. The development of the DLM program involved consultation with the Aboriginal Programs Advisory Group within the health services and the local ACCHS (which did not offer NSP or specialized sexual health services). It was important that the DLM program and mainstream health services be presented as an additional choice for Aboriginal clients and that consultations with the ACCHS focused on appropriate referral pathways between ACCHS and mainstream services. A key component of the start-up of the DLM program was to invite community Elders to visit the program and experience it even though they may not fall within the target group.

This paper uses data collected as part of an evaluation of the DLM pilot program (in relation to participant attendance in various aspects of the program), and qualitative data from interviews with DLM clients and staff to examine the acceptability of the program as the first step of a scalability assessment (defined as assessing effectiveness potential reach and adoption, alignment with strategic context and acceptability and feasibility) [2]. In particular, we examine the consistency of the intervention with the relevant strategies in HCV and STI program delivery, the resources (organizational, technical, human, and financial) required to deliver the intervention, the acceptability of the intervention to the target group and stakeholders, the potential for adaptability in other settings, and issues of reach and adoption of the intervention.

## Methods

Some data have already been presented in relation to preliminary outcomes of the DLM program from one site in the first 12 months of operation [20]. The current analysis extends this by including two sites over a longer period, providing data regarding the cascade of care from education to screening, and exploring the perceptions and experiences of staff and clients in the context of a scalability assessment. The two sites included in this evaluation are geographically located in bordering but separate areas governed by local health authorities. To avoid “double dipping” of the sites into the potential client pool, the sites have maintained strong collaborative relations with identified Aboriginal workers as “front-line” stafffor the DLM education component. This paper focuses on issues of process to inform scale up plans for future sites and ongoing development of implementation plans in existing DLM sites. This evaluation received approval from the human research ethics committees of the appropriate health services (those associated with Site 1 and Site 2) and the Aboriginal Health and Medical Research Council of NSW.

### Quantitative data

The DLM program operated 2 days per week at Site 1 from 29 April 2013 (2.5 years data available), and 1 day per week at Site 2 from 12 February 2015 (1-year data available). De-identified data on clients participating in the DLM program were obtained. Data included date of initial attendance, demographic characteristics (age, gender, suburb of residence), risk factors (injecting drug use), recruitment of others to the program (peer referral), and “cascade of care” progression (e.g., health education, screening, returned for results, treatment), as identified from incentive payment amounts. The overall numbers of Aboriginal and non-Aboriginal clients attending Site 1 sexual health clinic and numbers of occasions of service provided to Aboriginal and non-Aboriginal clients at Site 2 sexual health clinic were also obtained.

Comparisons of the proportion of Aboriginal clients (Site 1) and occasions of service provided to Aboriginal clients (Site 2) in the 12 months prior and 12 months post introduction of the DLM program were conducted using chi-square tests.

### Qualitative analysis

During 2015 and 2016, semi-structured telephone interviews were used to explore the perceptions and experiences of the DLM program among two key stakeholder groups: (i) health staff (including Aboriginal and non-Aboriginal workers); (ii) Aboriginal DLM participants. Staff were informed of the program evaluation and asked via their professional networks to volunteer for an interview. Interested participants approached a researcher not involved in the running of the DLM program and a time was scheduled for a telephone interview. Aboriginal participants were informed about interviews by the DLM program workers and referred on to a researcher if they were interested in participating. Due to the length of time that had passed between the beginning of the DLM program in Site 1 and the time of the evaluation, many DLM clients could not be reached by the program workers for invitation to an interview. The workers therefore invited clients who had current contact details or who were still engaged with the site.

A total of 13 interviews of around 45-min duration each were conducted via telephone with staff involved in the pilot program at these two sites. This sample included the majority of staff engaged with delivery of the DLM program. In addition, a total of 19 semi-structured interviews of around 15–30-min duration were conducted via telephone with Aboriginal clients of the DLM program (10 clients from Site 1 and 9 clients from Site 2). The sample size was sufficient to capture the complexity of program implementation across the two sites. Interviews with staff were conducted by a non-Aboriginal researcher (CT) and focused on: responses from Aboriginal communities and services regarding the program; how the DLM program operated within existing structures; strategies workers used to identify and solve issues; how the DLM program has been/could be embedded in routine policy and practice; and any unanticipated impacts of the program. Interviews with DLM clients were conducted by an Aboriginal researcher (VS) and explored the breadth and relevance of HCV and BBV prevention information, and clients’ level of comfort when attending the program sites.

All interviews were audio-recorded and transcribed verbatim. A thematic analysis [21] was conducted by two experienced qualitative researchers (CT, MH) and an Aboriginal research assistant (MW). All authors reviewed the preliminary findings. Findings of the qualitative evaluation of the DLM pilot program report the perceptions and experiences of staff members such as sexual health physicians, health education officers, Aboriginal peer-workers, NSP workers/educators, nurses, and clinical nurse consultants, as well as the experiences and perceptions of clients of the DLM program. A key aspect to inform implementation of interventions is to define the “active ingredients” which need to be applied with careful fidelity and those which can be modified to meet local needs and opportunities [22]. Further, a rich description of context is also required to enable implementation plans. The program logic that underpinned DLM identified Aboriginal staff and use of incentives as “active ingredients”. Thematic analysis of these data examined the assumptions of the program logic and the key active ingredients as well as exploring issues of context relevant to inform implementation plans.

## Results

### Quantitative data

A total of 677 and 55 Aboriginal clients attended Site 1 and Site 2 for health education, respectively. Demographic characteristics of DLM clients attending both services are presented in Table 1.

**Table 1:** Demographic characteristics of DLM clients

	Site 1 (*n* = 677) April 2013–Oct 2015 *N* ( %)	Site 2 (*n* = 55) Feb 2015–Feb 2016 *N* (%)
Age M (SD), range	33.45 (11.87), 14–91	38.62 (12.11), 18–64
Gender		
Male	320 (47)	33 (60)
Female	357 (53)	22 (40)
Area of residence		
Site 1 area	463 (68)	10 (18)
Site 2 area	85 (13)	41 (75)
Out of area	99 (15)	4 (7)
Not recorded/unknown	30 (4)	–
Injecting drug use*	196 (29)	15 (27)
Recruited other people to DLM (peer referral)	180 (27)	9 (16)

*History of injecting drug use was not a routine screening question. This information was gathered either through recognition of a DLM participant as an NSP client or through conversation

**Fig. 2:**
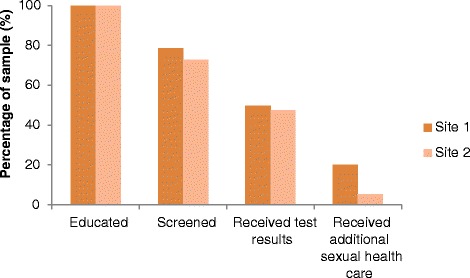
“Cascade of care” progression of Deadly Liver Mob clients. Note: Not all participants required additional sexual health care (e.g. treatment for sexually transmitted infections)

Figure 2 shows the “cascade of care” progression for DLM clients at both sites. Of those attending Site 1 for health education, 79% went on to attend screening, of which 50% returned to receive test results. Of those attending Site 2 for health education, 73% went on to attend screening, of which 48% returned for test results.

**Fig. 3:**
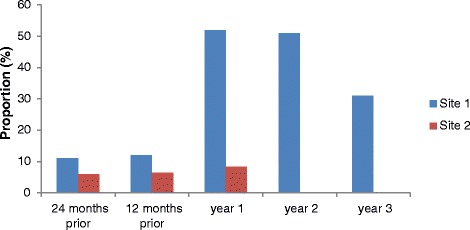
Overall proportions of Aboriginal clients (Site 1) and occasions of service provided to Aboriginal clients (Site 2) of the Deadly Liver Mob program. Note: Site 1 refers to % Aboriginal clients; Site 2 refers to % occasions of service provided to Aboriginal clients

The overall proportions of Aboriginal clients attending sexual health clinics for Site 1 and occasions of service provided to Aboriginal clients for Site 2 are presented in Fig. 3. For Site 1, the greatest proportion of unique clients attending the clinic (i.e., not repeat attenders) was 52% in the first year of the DLM program (April 2013 to April 2014). Between April 2011 and April 2015, the proportion of Aboriginal clients attending the clinic increased significantly from 11 to 52% (*p* < 0.001). This number dropped to 31% in 2016 or year 3 (*p* < 0.001). For Site 2, the proportion of occasions of service provided to Aboriginal clients between 2013 and 2015 (the DLM program was introduced in 2015) increased from 5.9 to 8.4% (*p* < 0.001).

### Qualitative data

DLM was typically well supported by staff and client participants. The DLM program logic identified that involvement of Aboriginal workers was central to the design. The qualitative data supported this assumption but provided a broader lens with which to understand this element of the program design and potential impacts of DLM. The main theme identified in these data was stigma: how stigma related to Aboriginality, HCV, and injecting drug use acts as a barrier to Aboriginal people’s engagement with information and health care services; and, how engagement increased when health-related stigma was minimised via attendance at the DLM pilot program. The qualitative data also supported the assumption that provision of incentives was a key ingredient. However, there were some strong and negative opinions, particularly from staff participants, which would be important to address in scale up plans. For DLM, key elements of context in these two sites were the capacity of the workforce and issues relating to the management of DLM especially in relation to navigating priorities across NSP and sexual health service delivery sectors.

#### A stigma-free health care and education program led by Aboriginal workers

Analysis of interview data highlighted the constraints that stigma places upon Aboriginal people’s access to health care services, and conversely, how Aboriginal people readily attend services where they do not feel negatively judged. The DLM pilot program was led by Aboriginal workers and provided an innovative model of a one-stop-shop, walk-in clinic for HCV education and harm reduction, BBV and STI screening and care that circumvented the stigma and discrimination experienced by DLM participants (many of whom inject drugs and/or live with HCV). Data from staff and clients demonstrated that a stigma-free, client-centred model of health care and education can significantly increase Aboriginal people’s access to and engagement with health-related services. Aboriginal staff of the DLM program were identified as key to ameliorating the potential impact of stigma in mainstream health services.

‘[I]t’s just nice to have someone on your level that is sort of – I don’t mean to be racist or anything like that, but this is not a white person who talks there with professional talk you know. The people there will have a yarn with you and sort of understand of where you are coming from.’ (Client, Site 2, #6)

‘[I]t’s very difficult for them to speak to somebody about it in a non-judgmental way, so they often live in silence in relation to their hepatitis C.’ (Staff #5)

‘I think it took off quicker than we imagined … from the kick start, we tapped into a couple of great networks very early and [the Deadly Liver Mob program] was novel and different and everyone was on a high, so our workers were particularly enthusiastic, as were we, and we could not have anticipated the uptake … and we’re still way in front if you look at the sexual health screening numbers, miles in front of where they were prior…’ (Staff #12)

The DLM pilot program’s education and health services were delivered in a friendly, culturally sensitive, non-judgemental environment, which made clients feel comfortable and was described as increasing the likelihood they would return to the service at a later date. In particular, the DLM program was seen as a gateway to sexual health awareness and engagement for younger Aboriginal clients.

‘And the people are so nice here, they make you feel – like if you’re wrong about something, they don’t make you feel stupid, which is what a lot of Indigenous people would have a problem with, because of education and that. I had problems with learning and that, but they don’t make you feel silly here. You feel like you’re equal. They explain it to you in the way that you understand.’ (Client, Site 1, #4)

‘I think that sexual health has such a stigma in that community … I think DLM was able to legitimize [Aboriginal] people getting a sexual health check-up, because I think to actually walk into a sexual health clinic, you are seen to be doing the wrong thing. “Why are you doing that for”, you know? Where it’s attached to some other type of care, or like getting some education … it may assist you to be empowered in other parts of your life and … you know [be seen to be] taking care of yourself …’ (Staff #7)

Overwhelmingly, staff gave a positive appraisal of the DLM pilot program and this was supported by statements from the clients. A large number of Aboriginal clients had been exposed, often for the first time, to health promotion education and BBV/STI screening. The co-location of NSP and sexual health services was said to have substantially improved Aboriginal clients’ access to information about HCV and safer injecting, BBV and STI screening, and referral for HCV treatment.

‘I’ve actually found out I can get rid of it [hepatitis C] and I’m going to try and start treatment, yes.’ (Client, Site 1, #11)

‘I thought it was absolutely amazing, because I knew nothing about what they were talking about and I was very informed. I liked it.’ (Client, Site 2, #5)

‘I thought it was good you know raising awareness of you know hepatitis and you know just harm minimisation and stuff like that you know. I thought it was good information.’ (Client, Site 2, #8)

#### Incentives

The provision of incentives was a key component of DLM but is not standard practice in health care delivery in Australia. Some staff expressed strong opinions about the use of incentives and questioned the ways in which these were used within DLM, potentially as “bribes” for attendance at a health service which clashed with the ethos of some staff participants. Some operational issues with vouchers were also raised including that storing and activating vouchers was sometimes difficult and required the development of a security process. On the other hand, the incentives were seen to play an important role in attracting clients to the pilot program and for opening discussions about sexually transmissible infections, safer injecting, HCV, and its treatment.

‘It’s not about the money. It’s about helping the Aboriginal community in my opinion. I mean, I wanted to come in here and just get better educated on it all and it’s been a great experience in my opinion.’ (Client, Site 1, #5)

Yeah, it’s a funny thing. I’m fairly – I personally don’t agree with [giving incentives], but as far as this group goes, I see that it can be a positive thing, so I don’t know. I’m happy to go with whatever. (Staff #10)

I keep on wondering like everybody else, would [the program] work or not, because I for one was not a big fan of incentives. I thought it was not the way to go, because it was just bribing people … but then again after it started and then it just took off, I thought, “Yes this is the way to go”. (Staff #9)

‘[I]t seemed that the benefits [of the pilot program] out-weighed the cost, and the cost was not big. You know, think about the money we’ve paid [for incentives] compared to the cost of treating someone if they went into hep C treatment … it feels like a no brainer … but I think for [Aboriginal] people, I think that they’ve got so many other things to contend with’ (Staff #12)

#### Workforce capacity

Staffing composition was said to be important for the success of a scaled-up program. Specifically, it was recommended that workers should comprise a mix of Aboriginal and non-Aboriginal people. Staff were often exposed to clients’ disadvantage and trauma, putting workers at risk of burn-out. The mental and physical health of workers requires monitoring and supporting through debriefing programs and ongoing primary care. The work place in which DLM operates requires mandatory training in Aboriginal cultural awareness. In addition to this, participants suggested that non-Aboriginal staff should be required to attend a pre-employment, awareness raising program to familiarize workers with the social disadvantage faced by Aboriginal communities and the impact that disadvantage has on clients’ health.

‘And you know you can’t help but be touched by the stories and even though I'm experienced in this area I would still feel, at the end of some days, I would just feel wiped out and not from the physical workload it’s the emotional side of it and hearing the stories and the level of disadvantage and the sadness in people’s lives and … the things that have gone on in their lives, and it just makes you realize even more you know, walk a mile in my shoes before you, you can never judge people can you?’ (Staff #8)

Aboriginal workers helped to recruit clients, greeted clients at reception, conducted the education interventions, provided information and advice to clients, and assisted with follow-up contact and care. Aboriginal workers reinforced an understanding among clients that this was a program administered by Aboriginal people, for Aboriginal people, with information and services relevant to Aboriginal communities. Aboriginal workers were particularly effective at establishing rapport with clients during the pilot program, and they facilitated the building of strong, trusting connections with local Aboriginal communities, which should be a prerequisite for establishing a scaled-up program. Aboriginal communities have diverse cultural conventions, for example, around making eye-contact, so Aboriginal workers are required to consult with communities about their specific needs.

‘[The staff] are on your level, they talk to you on your level and they don’t make you feel uncomfortable or they don’t get you impatient, you know what I mean? They don’t keep you there … they are very fast and very dedicated to their work, which is good.’ (Client, Site 2, #6)

‘I think it’s important to talk to the Elders in people’s area to find out what the culture is, whether it’s urbanized, whether it’s traditional … to understand what marks of respect and culturally appropriate steps to take with that client group.’ (Staff #6)

#### Management strategy

Strategies that were thought to increase program adoption and maximise program reach into Aboriginal communities were identified. Several practical suggestions for scale-up included the need for a steering committee and a co-ordinator to oversee the running of the program and to ensure that the DLM program has optimal impact by providing the full range of services. NSPs and sexual health services, although there may be some strategic and operational areas of common interest, are usually discrete entities within the Australian health care system, with different strategic priorities, workforce, operational policies, procedures, and clientele, so it is necessary to develop strategic approaches to managing and promoting innovative co-located services within Aboriginal communities. For example, as a result of logistical barriers and budget restrictions, one sexual health service operating in the DLM pilot program did not provide the full range of tests required to diagnose chronic HCV infection.

Careful planning and consideration is required to identify geographic areas where there is strong Aboriginal community connections in order to maximise program adoption and reach. The pilot program evaluation indicated that it is useful to locate DLM programs close to where Aboriginal people meet to minimise the effort required for participants to attend DLM and to make it easier for DLM workers to build relationships with the community. Similarly, ongoing community consultation with Aboriginal organisations, such as ACCHS, was recommended as an important management strategy to ensure continuing smooth operation of the program.

‘[W]e started off with the optimal kind of site there at [western suburb], we were close to, across the road from the major shopping centre, right next to the transport hub, near the methadone clinic, not far from the drug and alcohol services, right next door to the sexual health service and right in a very, very high density Aboriginal population sort of heartland, so you know, we had a great combination of factors there in setting up and we had some really good community, some great community relationships there.’ (Staff #13)

A walk-in, one-stop shop model of care, with flexible appointments, short wait times, and child-friendly waiting areas was described as the most effective model for increasing community adoption and the reach of a scaled-up program. In regard to the make-up of DLM services, ideally, these were thought to include primary health care, HCV PCR testing, HIV and HBV antibody testing, liver disease assessment, access to HCV treatment using the direct acting antivirals (DAAs), pap smear, and a variety of sexual health tests. In the era of DAAs and efforts to increase HCV treatment uptake, a scaled-up DLM program has the potential to give clients direct access to effective HCV treatment. Finally, clients’ low literacy levels need to be considered in the design of the resources they receive in their DLM package and staff should be prepared to assist clients with low literacy to fill out paper work.

‘… I think a one stop shop [is the best model], so have the social worker in there, have the sexual health all in the one spot if you can possibly have it … because traditionally … people go to NSP’s, go to methadone clinics, they don’t travel very far if it’s not within that service. Getting them to go to a [mainstream health] service is a lot more difficult.’ (Staff #11)

## Discussion

This evaluation presents evidence to consider in the broader implementation, or scale-up, of the DLM program. Significant numbers of Aboriginal people were engaged in the program, acceptability of the program was high among staff, and clients and additional resources required to deliver the program (relating to incentive payments) were modest. Some issues should be clarified in development of an implementation plan: the relationship of DLM with the relevant strategies for HCV and STIs, better support for the workforce to understand the role of incentive payments, additional resources to induct and support workers, and methods to “manage up” in explaining the impact and role of DLM over time.

One purpose of conducting assessments of scalability is to examine the influence of strategy, policy, and operational issues [23]. The combination of HCV and sexual health/STI activities within DLM posed issues in relation to strategic concerns. The NSW HIV [24], Hepatitis C [25], and STI [26] strategies are not completely complementary or aligned with the DLM activities. That is, although Aboriginal people are a priority population for these strategies, the STI strategy does not include reference to HCV. Sexual health services are acknowledged in the hepatitis C strategy as sites of assessment and care. However, sexual health services are one of many types of services included and there is no further elaboration on models of care or strategic referral pathways for HCV care in sexual health services. This can create situations in which HCV screening is not ‘core-business’ of sexual health services participating in DLM programs. For example, staff may not be familiar with issues experienced by people who inject drugs and the full range of tests required for HCV may not be provided, or within the budget of, sexual health services as was apparent in one DLM pilot site. Hence, the set-up of future programs should carefully investigate this issue and develop protocols to allow the “one stop shop” approach that is at the heart of the DLM program to provide full HCV screening and HCV treatment in sexual health clinics.

This slight strategic misalignment also has implications for characterising the DLM target group. Although DLM is framed around engagement of people with, or at risk of, HCV (who may be of any age), sexual health services see people under 35 years as their key target group. There is arguably a third target group for DLM: Aboriginal community members, irrespective of risk factor history or demographics. This third group is important for disseminating information about HCV and reducing stigma associated with it, and in generating community understanding, acceptance, and encouraging participation in DLM. It is recommended that the DLM sites set clear and appropriate goals regarding the extent to which they will engage with the target groups in each component of the program. For example, all Aboriginal community members who seek education will be accommodated at all stages of the program, regardless of history of injecting drug use; all Aboriginal community members agreeing to sexual health screening will be accommodated within the first period of the program (to be determined) to build trust, rapport, and understanding with community and community leaders; after this period, sexual health screening will be targeted to people 35 years and under who are sexually active; and, Aboriginal people who inject (or have injected) drugs remain the priority target population throughout. It is important that the inclusion of other groups does not exclude or dissuade the involvement of the priority group.

Financial incentives are increasingly recognized as valid strategies for encouraging the uptake of public health measures. Although financial incentives for community members to improve health-related behaviours have had a limited role in the Australian setting, they have been widely used internationally, particularly in lower and middle income countries [27, 28], have been demonstrated to be an effective means of changing behaviours and increasing uptake of interventions [29, 30] and have been discussed as an “ethical” public health approach [31]. In the area of sexual health, financial incentives have been shown to increase testing rates for both HIV and Chlamydia [32–34] among people at higher risk. A model for Aboriginal sexual health in Australia has been established by the Young Persons Check, which has been conducted annually by external clinical teams visiting for a short period of time in a number of communities in Cape York and offering a $20 phone voucher to young people who agree to have an STI test. The program has achieved testing coverage in the range 65–80%, sustained over 3 years [35].

In DLM, incentives played a clear role in encouraging decisions to participate. Given the mistrust of mainstream health services that can underpin Aboriginal Australians’ decisions about health care engagement, the provision of an incentive was also a clear signal that the program could offer something of tangible benefit to Aboriginal people. Understanding the perspectives of the Aboriginal community and the evidence-base regarding incentive payments should be key elements of staff training regarding the incentive-based nature of the DLM program.

Ways in which DLM staff could be supported were apparent from these data and included, in addition to any Aboriginal cultural sensitivity training offered by the workplace, the need for staff members to understand the role that social disadvantage plays in clients’ lives and how disadvantage affects clients’ health [36]. It was also recommended that staff have an extensive knowledge of injecting drug use, HCV infection, sexual health, treatments, and other health issues. Due to the emotionally challenging work at DLM programs, debriefing for staff members, particularly Aboriginal workers, is required.

The data from participant attendance draws attention to the need to ensure that all stakeholders understand what constitutes success in the DLM program. These data show very significant increases in attendance of Aboriginal people in the first year of Site 1. Attendance remains high, but lower, in the subsequent years of DLM. DLM sites need to ensure that funders and health managers understand patterns of DLM attendance and have appropriate expectations of community engagement and activity. For example, rates of attendance at DLM may decline over time, but remain significantly higher in relation to baseline. This pattern is expected and appropriate as saturation is reached within the community surrounding the DLM site. Hence, establishment of an appropriate and rigorous baseline measurement is essential.

There are limitations in the data collected during this pilot study which effect the generalizability and transferability of findings to other contexts. In particular, the staff interview data was relatively richer than the narratives provided by DLM client participants. This could be due to varying education levels (as alluded to by participant #4) and issues of stigma and power (Aboriginal people having relatively fewer opportunities to express their opinion). Further, there were challenges faced by the DLM program workers in inviting Aboriginal clients to participate in interviews, due to outdated contact details. The interview sample of Aboriginal clients is therefore limited to those who had not changed their contact details or who were still connected to the site, thus may be considered more engaged with the service. The findings are drawn from an exploratory analysis of a pilot program and as such the qualitative data are limited to a descriptive analysis of top-level themes. Despite this, these themes highlight and illustrate the major barriers and facilitators to engagement with the DLM program. These findings, while limited, are important to acknowledge when planning future studies of Aboriginal health care engagement, both in regard to the DLM program, and more generally, and when considering the scale-up and roll out of this innovative model of care for Aboriginal people.

Finally, the larger context of this program needs to be considered in the central role of a stigmatized community as well as stigmatised and illegal practices. It was an important aim of this program that DLM did not perpetuate stigma and worked to reduce stigma against Aboriginal people who inject drugs and who live with HCV. Aboriginal people who inject drugs are among the most stigmatised population groups in Australia, and their unequal status often becomes evident when they access health services [10, 37, 38]. The socio-cultural, economic, and political values that underpin mainstream health services, such as the expectation of compliant health-consumer behaviour (e.g., attending appointments on time, returning for test results), can be difficult to navigate given the social and economic challenges that many Aboriginal people experience, such as concurrent family violence, addiction, chronic illness, homelessness, and poverty [39]. Stigmatization is a social process that produces, perpetuates, and legitimizes social inequality [40, 41]. Aboriginal people who inject drugs may be labelled by health workers and others as an infectious risk, mentally unstable, untrustworthy and, importantly, someone who caused their own ill health and therefore are less (or not) deserving of care [42, 43]. Social inequality has material as well as psychological effects on stigmatized individuals and groups; on the one hand, the stigmatizing process leads to an internalizing of shame and on the other hand it creates a barrier to patients’ future engagement with mainstream health services [44]. That the DLM program was strongly endorsed by clients as non-stigmatizing, and that they could nominate a number of benefits to the program, underlines the importance of measures that reduce the experience of fear of stigma for Aboriginal DLM clients as key ingredients of the program to maintain in future implementation.

## Conclusion

Delivering effective and acceptable care for Aboriginal people in Australia is a challenge in all areas of health. This challenge becomes more complicated when sensitive and stigmatized issues of sexual practice and injecting drug use are implicated in the health conditions of interest. While ACCHS remain the cornerstone of delivery of primary care for Aboriginal people, publicly-funded “mainstream” services have a key role to play for BBV and STIs given their specialist services and as an alternative option for service access among this population. However, the capacity of publicly funded services to engage Aboriginal people has been limited over many years, with very low rates of participation in these services. How to effectively engage Aboriginal people remains a complex question that requires an effective and engaged partnership of policy makers, researchers, service managers, and front-line workers to address. The DLM program shows promise in this regard, and the potential impact of this program should be examined when implemented carefully, and at scale.

## Abbreviations

DLM: Deadly Liver Mob
HCV: Hepatitis C virus
NSW: New South Wales
STIs: Sexually transmissible infections
